# Decoding the pathological and genomic profile of epithelial ovarian cancer

**DOI:** 10.1038/s41598-024-80030-z

**Published:** 2024-11-19

**Authors:** Rim Rejaibi, Arnaud Guille, Maroua Manai, Jose Adelaide, Emilie Agavnian, Aida Jelassi, Raoudha Doghri, Emmanuelle Charafe-Jauffret, François Bertucci, Mohamed Manai, Karima Mrad, Lamia Charfi, Renaud Sabatier

**Affiliations:** 1 Pathology Department, Salah Azaiez Institute, Tunis, 1006 Tunisia; 2 Biology Department, Laboratory of Mycology, Pathologies and Biomarkers (LR16ES05), Faculty of Sciences of Tunis, University of Tunis El Manar, Tunis, 2092 Tunisia; 3 Laboratory of Predictive Oncology, Centre de Recherche en Cancérologie de Marseille, CRCM, Inserm UMR1068, CNRS UMR7258, Institut Paoli-Calmettes, Aix Marseille Université U105, Marseille, France; 4 Centre de Recherche en Cancérologie de Marseille, ICEP Platform, CRCM, Institut Paoli-Calmettes, Inserm UMR1068, CNRS UMR7258, Aix Marseille Université U105, Marseille, France; 5 Laboratory of Transmission, Control, and Immunobiology of Infections, LR11IPT02 (LTCII), Tunis-Belvédère, Pasteur Institute of Tunis, University of Tunis El Manar, Tunis, Tunisia; 6 Laboratory of Precision medicine personalized medicine and oncology investigation, Salah Azaiez Institute, Tunis, Tunisia; 7 CRCM, Inserm, CNRS, Institut Paoli-Calmettes, Epithelial Stem Cells and Cancer Lab, Equipe labellisée LIGUE contre le cancer, Aix-Marseille Université, Marseille, France; 8 Department of Medical Oncology, Institut Paoli-Calmettes, Marseille, France

**Keywords:** Ovarian cancer, Tissue micro-array, Copy number alterations, Survival., Cancer, Genetics, Immunology, Molecular biology

## Abstract

Ovarian cancer (OC) is one of the most common cancers in women, with a high mortality rate. Most of published studies have been focused on Caucasian populations, with the need to explore biological features and clinical outcomes of patients from other ethnicities. We described clinical outcome (progression-free survival and overall survival) and biomarkers associated with survival in a cohort of patients with OC from Tunisia. Using immunohistochemistry, we assessed the expression of 14 proteins known to be altered in OC in a cohort of 198 patients. We explored the correlation between protein expression and copy number alteration (CNA) profiles. FIGO stage, menopausal status and mismatch repair deficiency were associated with survival. *ERBB2* amplification was correlated with high ERBB2 expression (OR = 69.32, *p* = 4.03 E-09), and high PDL1 expression was associated to *CD274* amplification (OR = 4.97, *p* = 5.79 E-2). We identified a correlation between survival and exposure to two CNA signatures (MAPK pathway and BRCA-related homologous recombination deficiency). Moreover, Gama-H2AX protein expression was correlated with exposure to a genomic signature associated with homologous recombination deficiency. We observed that OC clinical and pathological characteristics of these patients from Tunisia were similar to those of Caucasian patients. We identified frequent CNA in this population that need to be confirmed in other sets from Africa.

**Supplementary Information:**

The online version contains supplementary material available at 10.1038/s41598-024-80030-z.

## Introduction

Ovarian cancer (OC) is one of the most common cancers in women, with a high mortality rate. There are an estimated 239,000 new cases of ovarian cancer each year, representing 152,000 deaths worldwide^1^. Malignant epithelial tumors of the ovary represent more than 90% of OC^2^. They develop from the surface covering of the ovaries and the adjacent stroma or the fallopian tube. Unlike other ovarian cancers that typically affect young women, such as germ cell or sex cord tumors, these tumors occur mainly after menopause with a peak frequency between the 5th and 6th decades^3^. They also represent the most lethal form of gynecological cancer due to late diagnosis (more than 70% at stages III and IV of the FIGO classification) and the high rate of recurrence^4^. Indeed, despite standard treatments based on maximal cytoreductive surgery and chemotherapy combining platinum salts and paclitaxel, with more than 70% objective response, the overall 5-year survival rate remains close to 30%^5^. The pathological and clinical factors currently used to guide therapeutic management (age, FIGO stage, histological grade, resectability) do not seem to faithfully reflect the underlying biological evolutionary potential of these cancers^2^.

Some studies suggest that Asian and Caucasian populations may have different features, such as a higher incidence of clear-cell carcinoma in Asia^6^. In Tunisia, according to the cancer register for the northern region of Tunisia, 594 cases of ovarian cancer were recorded during the period 2010–2014. The mean age was around 54 years (54.3 ± 13.0 years), with 65.4% of cases aged 50 years and over. The standardized incidence rate was 4.3/100,000. This cancer ranked 7th among women. African countries show intermediate to low absolute mortality rates but high Mortality/Incidence ratios. This could be attributed to late diagnosis and a lack of access to adequate treatment, including sophisticated surgeries and chemotherapy regimens^7^. Moreover, little is known about the biological and genomic characteristics of OC from Africa^8,9^. Despite advances in the treatment of OC over the last few decades, the survival of African patients with OC remains poor. As a result, new markers and therapeutic targets need to be identified to improve treatment and survival in this population. Therefore, this study aimed to identify clinical and pathological features as well as recurrent copy number alterations (CNA) in a large cohort of Tunisian patients with OC.

## Results

### Demographics and pathological features

Among 198 patients treated at SAI between 2000 and 2018, the median age was 56 years (range 21–90). All demographics and clinicopathological data were summarized in Table 1. A third of the patients were premenopausal, and 67% were postmenopausal. Tumors were mostly diagnosed at FIGO stage III-IV (80.7%). As expected, the most frequent histological subtype was serous in 168 patients (85.2%), with high-grade serous carcinoma (HGSC) in 158 cases (80.6%) and low-grade serous carcinoma (LGSC) in 9 cases (4.6%). Additionally, 5.1% were clear-cell, 4.1% were endometrioid, 3.5% were mixed, and 2% were mucinous. More than 91% of patients had high-grade tumors, 49.2% patients underwent optimal debulking surgery, and 83.5% received neoadjuvant/adjuvant platinum-based chemotherapy. Due to the lack of access to innovative drugs, they have yet to receive bevacizumab.

**Table 1:** Baseline clinical and pathological characteristics.

Histopathological features (*N* = 198)		*n* (%)
Age		56 (21–90)
FIGO stage	Stage I	16 (8.9%)
	Stage II	19 (10.4%)
	Stage III	135 (74.2%)
	Stage IV	12 (6.6%)
	NA	16
Grade	High	179 (91.3%)
	Low	17 (8.7%)
	NA	2
Histological subtype	Serous (All)	168 (85.2%)
	HGSC	158 (80.6%)
	LGSC	9 (4.6%)
	Clear cell	10 (5.1%)
	Endometrioid	8 (4.1%)
	Mixed	7 (3.5%)
	Mucinous	4(2%)
Menopausal status	postmenopausal	110 (67.5%)
	premenopausal	53 (32.5%)
	NA	35
Surgery	optimal	97(49.2%)
	incomplete	43(21.8%)
	NA	8
Neoadjuvant/adjuvant chemotherapy	Yes	142 (83.5%)
	No	28 (16.5%)
	NA	28
Progression-free survival	Median, months (95% CI)	30.0 (18.0;48.0)
	24 m-PFS rate	58% 95CI (47.9–69.9)
Overall survival	Median, months (95% CI)	Not reached
	24 m-OS rate	81% 95CI (73.6–89.5)

NA : not available ; HGSC : High Grade Serous Carcinoma ; LGSC : Low Grade Serous Carcinoma ; 95CI: 95% confidence interval

**Fig. 1:**
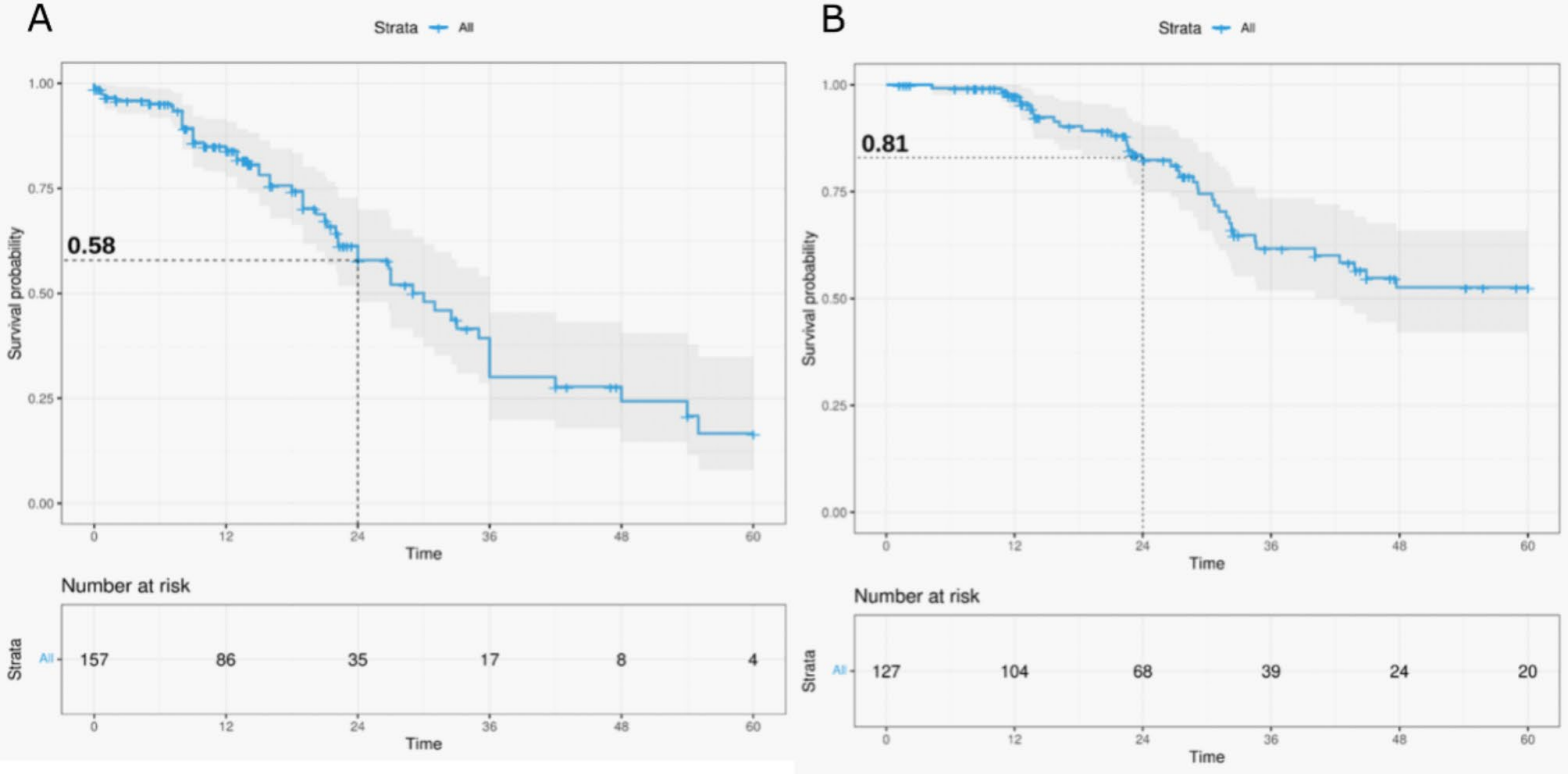
Kaplan-Meier curve for progression-free survival (**A**) and overall survival (**B**).

The median follow-up was 35 months. Sixty-seven patients presented a disease progression during follow-up, and 20.8% died. The median PFS was 30.0 months (95CI [18.0–48.0]), and the 2-year PFS rate was 58% (95CI [47.9–69.9]) (Fig. 1A). Median OS was not reached, probably due to the short follow-up. The 2-year OS rate was 81% (95CI [73.6–89.5]) (Fig. 1B).

## Protein expression profile

The expression of 14 proteins was measured in all 198 ovarian carcinoma samples (Table 2). 63% displayed P53 expression classified as a P53 abnormal profile. Some tumors expressed PD-L1 (37.3%) or EGFR (16.1%). Most tumors were estrogen receptor-positive (80.7%), 16% had low PTEN expression. MMR deficient (MMRd) profile status was correlated with histotypes (*p* = 0.04). MMRd profile was found in 3.68% of HGSC, 11.11% of clear cell carcinoma, 28.57% of endometrioid carcinoma, 16.67% of mixed carcinoma, and 0% of mucinous carcinoma (Sup Table S1).

**Table 2:** Proteins expression in ovarian carcinoma.

Protein	Threshold	Low expression, *N* (%)	High expression *N* (%)	NA
P53	90%	P53 Wild type54 (37%)	P53 abn92(63%)	7
Estrogen Receptor	10%	33 (19.3%)	138 (80.7%)	26
Progesterone Receptor	10%	129 (66.5%)	65 (33.5%)	3
PD-L1	1%	121 (62.7%)	72 (37.3%)	4
PTEN	0%	31 (16%)	163 (84%)	3
p16	70%	93 (48.7%)	98 (51.3%)	6
EGFR	0%	162 (83.9%)	31 (16.1%)	4
PMS2	10%	2 (1%)	189 (99%)	5
MSH2	10%	1(0.5%)	192(99.5%)	4
MSH6	10%	3 (1.5%)	190 (98.5%)	4
MLH1	10%	3 (1.56%)	189(98.4%)	4
ERBB2	Score 0/1 vs. score 2/3	177 (91.2%)	17 (8.8%)	
EZH2	15%	98 (50.5%)	96 (49.5%)	3
Gamma-H2AX	10%	109 (56.8%)	83 (43.2%)	5

N : number ; NA : not available ; abn : abnormal

## Correlation of protein expression with clinicopathological features in each histotype

After histotype stratification, we assessed correlations between the protein expressions and the clinicopathological features. In Supplementary Table 2, in the HGSG histotype, we showed a significant correlation between age and PR, P16, and PMS2 protein expressions (*p* = 1.52E-02,*p* = 1.73E-02, and *p* = 3.94E-02, respectively). In the HGSG histotype, the Ca125 was correlated with ER and ERBB2 (*p* = 4.07E-02 and *p* = 4.92E-02, respectively). Furthermore, metastatic lymph node status was correlated with P53, PDL1, and PTEN protein expression (*p* = 4.36E-02,*p* = 3.87E-02, and *p* = 2.98E-03, respectively). In clear cells carcinoma, only ER protein expression was correlated with the macroscopic tumor residues (*p* = 4.76E-02), and only the stage was correlated with ER expression in endometrioid carcinoma histotype (*p* = 2.86E-02). No other correlations were found in the mucinous and mixed carcinoma histotypes.

Furthermore, MMR deficient (MMRd) status was found correlated with histotypes (*p* = 4.64E-02). MMRd profile was found in 3.68% of HGSC, 11.11% of clear cell carcinoma, 28.57% of endometroid carcinoma, 16.67% of mixed carcinoma, and 0% of mucinous carcinoma (Sup Table S1).

## Genomic characterization of 198 ovarian tumor samples by CGHarray

**Fig. 2:**
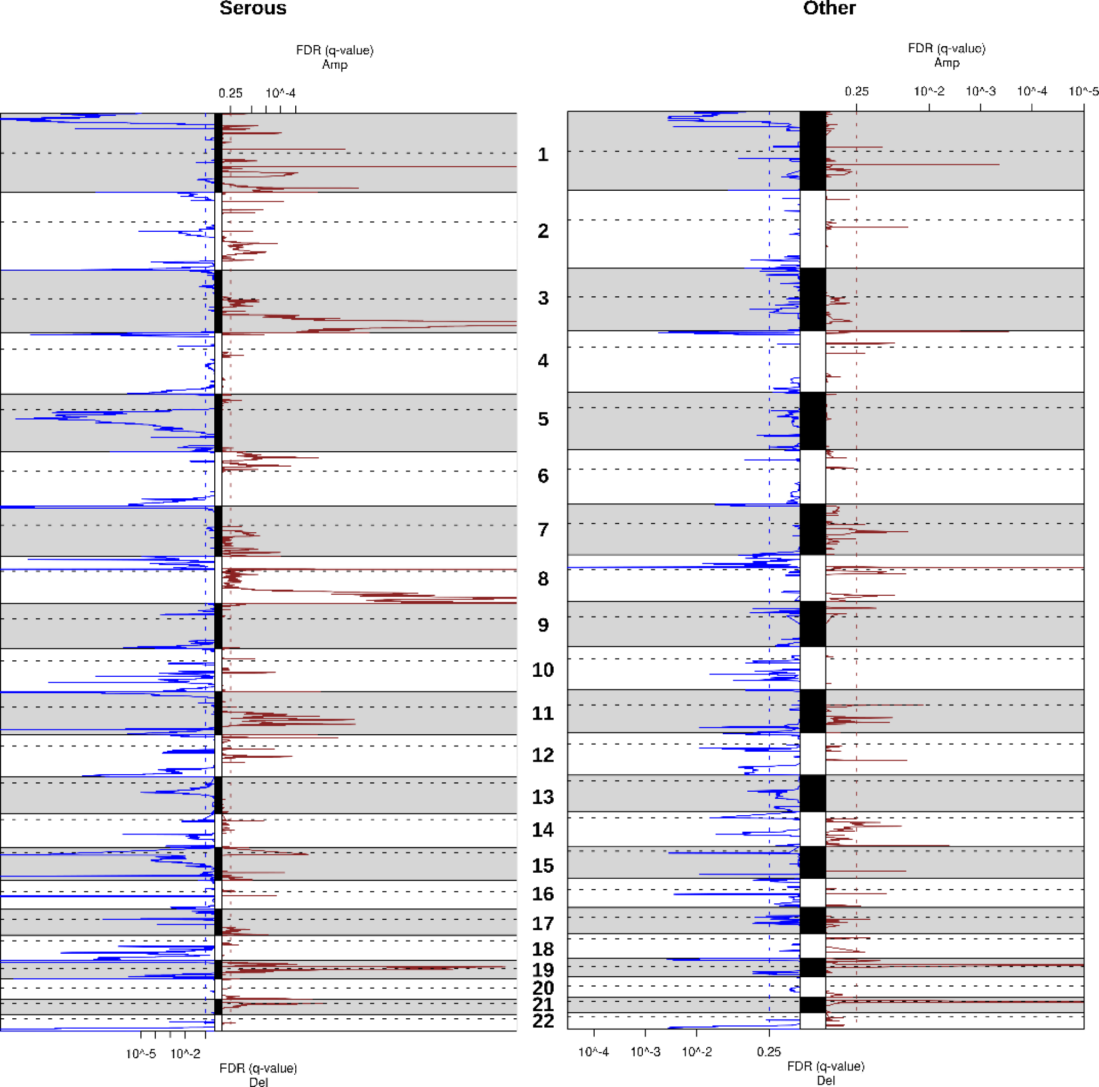
Copy number alterations profiles of EOC tumors. Frequency plot of recurrent copy number alterations was identified in OC tumors using the GISTIC algorithm. Frequencies of gains (left) and losses (right) are plotted due to chromosome location. X-axis: top = log–scale ratio; bottom = *q*-values. Left hand-side panel represent CNV analysis in high-grade serous tumor. Right hand-side panel represents CNV analysis in samples of other pathological subtypes.

The genomic profiles of 198 samples were established by aCGH, as previously reported^10,11^. CNA analysis showed a high level of genomic instability (Fig. 2). Frequently altered regions with low and high levels of amplification or loss in the 198 tumors were defined using the GISTIC algorithm. GISTIC analysis revealed 1233 amplified genes and 2973 lost genes. The ten regions most frequently gained were on the chromosome arms 8p11.22, 1q24.2, 19p12, 8q24.21,3q26.2, 21q11.2, 8q24.3, 12p13.31, 1p13.2, and 3q26.31. The most frequently lost regions were localized on 8p11.22, 19q13.4, 19p13.3, 16q12.1, 22q13.33, 1p36.13, 15q11.2, 15q25.3, 11p15.5, and 11q23.3.

**Fig. 3:**
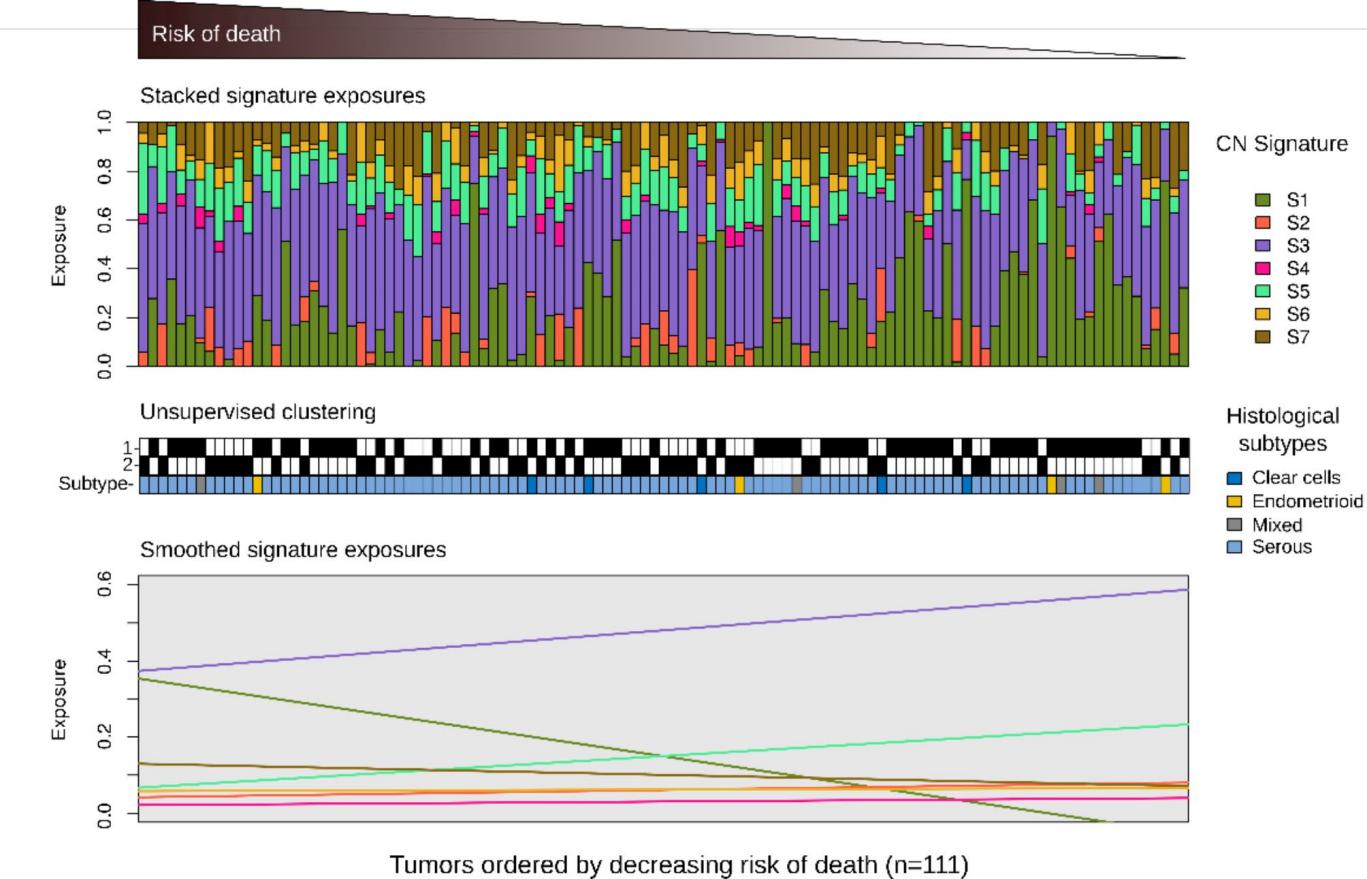
Association of survival with copy-number signatures. Upper panel: Stacked barplots show CNA signatures exposures for each patient. Patients were ranked by risk of death estimated by a multivariate Cox proportional hazards model stratified by age and cohort, with CNA signature exposures as covariates. Lower panel: Linear fit of signature exposures ordered by risk predicted by the Cox proportional hazards model.

We next explored the association between individual CNA signature exposures and overall survival using a combined dataset of 111 diagnostic samples with clinical outcomes^12^. As shown in Fig. 3, the three most represented signatures were signature 1 (MAPK pathway), signature 3 (BRCA-related homologous recombination deficiency), and signature 7 (non-BRCA-related homologous recombination deficiency). We observed a trend between signature 1 or signature 3 exposure and overall survival. As shown in Supplementary Table S1, after stratifying the histotypes, the s1, s3, s5, and s7 signatures remains associated to histotypes (*p* = 4.87E-05,*p* = 1.5E-03,*p* = 9.09E-03, and *p* = 5.7E-04, respectively).

**Fig. 4:**
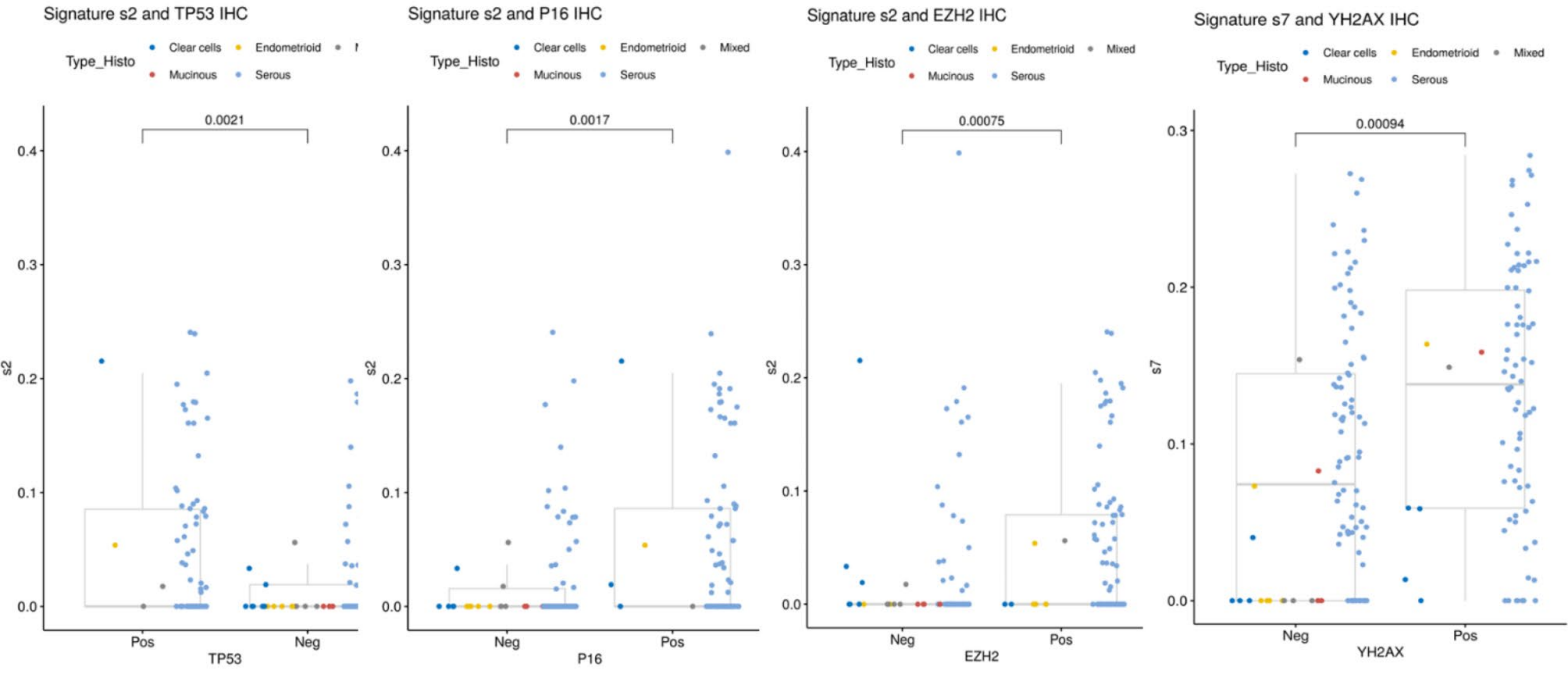
Association between IHC expression and CNA signatures (only statistically significant correlations are shown here, full data are available in Supp Figure S4). Dots color represents the pathological subtype of each tumor: light blue = serous, dark blue = clear cell, red = mucinous, orange = endometrioid, grey = mixed.

Furthermore, some proteins were correlated with CNA signature exposures (Fig. 4). At the FDR threshold < 0.1, p53abn profile, p16, and EZH2 expression were correlated with the s2 signature (Tandem duplication, CDK12 mut). The Gamma-H2AX protein was significantly correlated with exposure to the s7 signature (non-BRCA-related homologous).

## Correlations between protein expression and genes copy number alterations

PDL1 expression was associated with amplification of CD274 (OR = 4.97, 95CI [0.63-43], *p* = 5.79 E-2). We also observed a correlation between *ERBB2* amplification and ERBB2 protein expression *(OR = 69.32*,* 95CI [7.6–3849]*,* p * = 1.67E-05). P16 loss of protein expression was associated to deletion of CDKN2A (OR = 0, 95CI [0-1.2],*p* = 1.04 E-02).

Gene losses concerning CD274 (OR = 0.40,95CI [0.14–0.79], *p* = 1.48E-02), PTEN (OR = 0.4,*p* = 3.94E-02), CDKN2A (OR = 0.28,95CI[0.12–0.6],*p* = 3.78E-04), and *ERBB2 (OR = 0.16*,*95CI[0.037–0.63]*, *p* = 1.62E-03) were correlated to lower expression of the corresponding proteins (Sup Table S3). By correlating *ERBB2* amplification with the different histotypes, it was found predominantly amplified in HGCS (*p* = 1,58E-03) (Sup Table S4).

We compared the degree of CNAs leading to amplification/deletion in the regions found in GISTIC to protein expression in IHC in the 198 tumors. Using *p* < 0.05 and FDR < 0.25 as thresholds, overexpression of Gamma-H2AX was linked to amplification in 8q24.3 and 8q24.21. High expression of p16 was linked to amplification in regions 3q26.1, 3q26.2 including *MECOM, EGFEM1P*, and *hsa-mir-551b*, and 3q26.31, which was also linked to high expression of P53. This region includes *ECT2*,* GHSR*,* TNFSF10*,* NCEH1*,* and FNDC3B.* High expression of PTEN was associated with amplification in 8q24.21. High expression of EZH2 was linked to amplification of *MAGI3* (1p13.2) (Sup Table S5).

### Correlations between clinicopathological features and outcome

As mentioned above, the 2-year PFS rate was 58% (95CI [47.9–69.9]), and the 2-year OS rate was 81% (95CI [73.6–89.5]) in our cohort. Poorer PFS tended to be correlated with advanced FIGO stage *(HR = 2.0*,* 95CI [0.94–4.40]*,*p* = 0.07) and residual disease after debulking surgery (HR = 1.6, 95CI [0.92–2.90],*p* = 0.09) (Table 3). None of the proteins explored in this study was associated with PFS, even if we observed a trend (HR = 1.7, 95CI [0.99–2.9]) for Gamma-H2AX (Table 4).

Overall survival was significantly correlated with menopausal status (HR = 2.3, 95CI [1.1–5.1],*p* = 0.044) and low MSH2 expression (HR = 0.1, 95CI [0.01–0.82],*p* = 0.03) (Table 4). OS tended to be poorer in patients with advanced stage *(HR = 3.1*, 95CI [0.91-10.0],*p* = 0.072) (Table 3).

**Table 3:** Correlation between clinicopathological features and survival.

	Progression-free survival 24 m-PFS rate : 58% 95CI (47.9–69.9)			Overall survival 24 m-OS rate: 81% 95CI (73.6–89.5)		
Feature	N	HR (95% CI)	p-value	N	HR (95% CI)	p-value
Postmenopausal	157	1.3 (0.7–2.3)	0.45	111	2.3 (1-5.1)	**0.04**
FIGO Stage III-IV vs. I-II	169	2 (0.94–4.4)	0.07	113	3.1 (0.91-10)	0.072
Grade high vs. low	184	1.3 (0.56–3.1)	0.52	120	1 (0.25–4.3)	0.96
High-grade serous vs. Others	184	1.1 (0.54-2)	0.88	120	0.78 (0.32–1.9)	0.58
Residual disease after surgery vs. Complete debulking resection	139	1.6 (0.92–2.9)	0.09	98	1.4 (0.73–2.7)	0.30

Significant values are in bold.

N: Number ; HR: Hazard Ratio ; 95CI: 95% confidence interval ; FIGO: International Federation of Gynecology and Obstetrics

**Table 4:** Correlation between proteins expression and survival.

	Progression-free survival 24 m-PFS rate : 58% 95CI (47.9–69.9)			Overall survival 24 m-OS rate: 81% 95CI (73.6–89.5)		
Feature	N	HR (95%CI)	*p*-value	N	HR (95%CI)	*p*-value
P53	177	0.93 (0.53–1.6)	0.78	115	0.59 (0.31–1.1)	0.11
ER	158	1.5 (0.66–3.3)	0.35	109	0.44 (0.18–1.1)	0.07
PR	181	0.85 (0.49–1.5)	0.57	119	0.73 (0.37–1.4)	0.34
PDL1	180	0.99 (0.58–1.7)	0.98	118	0.58 (0.3–1.1)	0.11
PTEN	181	1.7 (0.71–3.9)	0.24	118	1.5 (0.46–4.9)	0.50
p16	178	1.2 (0.7-2)	0.52	116	0.79 (0.42–1.5)	0.46
EGFR	180	1.3 (0.53-3)	0.60	118	1.1 (0.43–2.8)	0.85
PMS2	179	2.6e + 07 (0-Inf)	0.99	117	2.5e + 07 (0-Inf)	0.99
MSH2	180	33e + 05 (0-Inf)	0.99	118	0.1 (0.01–0.82)	**0.03**
MSH6	180	2.6 (0.35-19)	0.35	118	0.15 (0.02–1.2)	0.07
MLH1	180	3,400,000 (0-Inf)	0.99	118	1.0 (0-Inf)	0.99
ERBB2	180	1.7 (0.4–6.9)	0.48	118	2.6 (0.81–8.7)	0.11
EZH2	181	0.99 (0.58–1.7)	0.98	118	0.79 (0.42–1.5)	0.46
Gamma-H2AX	179	1.7 (0.99–2.9)	**0.056**	117	0.85 (0.46–1.6)	0.61

Significant values are in bold.

N: Number ; HR: Hazard Ratio ; 95CI: 95% confidence interval

## Discussion

To our knowledge, we describe in this paper the first study exploring clinical, pathological, and genomic features of patients from North Africa diagnosed with OC. Clinical characteristics were close to those of other series in the same period. IHC analysis of 14 proteins of interest showed frequent P53 alteration, hormone receptor expression, and low rates of PTEN loss, MMR deficiency, and ERBB2 overexpression. As expected, tumors in our set displayed a high level of genomic instability. The prognosis tended to be correlated with the FIGO stage and the achievement of complete cytoreductive surgery. No significant prognostic correlation with protein expression could be identified.

The demographics of patients included in our set were similar to another Tunisian cohort of 104 patients with advanced OC treated between 1994 and 2004^8^. We observed similar rates of advanced FIGO stage, optimal debulking surgery achievement, and first-line chemotherapy regimen. Our cohort’s 2-year PFS and OS rates were 58% and 81%, respectively. The 2-year OS rate of 81% was higher than in the previous set (57%). This discrepancy may be explained by the lack of stage I-II cases in this cohort and our study’s later inclusion period (2000–2018), with improvements in the whole OC management, including improvement in surgical procedures, chemotherapy regimen, and later lines of systemic therapies.

Most ovarian epithelial tumors express estrogen and progesterone receptors^13^. Our IHC results confirm these findings, with 80.7% overexpression of the Estrogen Receptor. P16 is a tumor suppressor protein known as cyclin-dependent kinase inhibitor 2 A (CDKN2A). Abnormal (low) p16 expression has been associated with poor prognosis in OC^14^. In this study, *p16* altered CNA profile was observed in 98 cases (51.3%). We observed similar rates of p16 loss in our IHC study but no impact on patients’ survival. Even though most OC with germline mutation are due to alterations in *BRCA1/2*, some are also associated with Lynch syndrome. Lynch Syndrome results from deleterious constitutional mutations in MMR genes^15^. In a recent study including 478 OC, Faune et al. showed a higher percentage of MMRd in endometrioid carcinoma (23%), which was in accordance with our results of 28.6% of MMRd^16^. Concerning the clear cell carcinomas, Jensen et al. found 12.5% of MMRd similar to MMRd status in our study with 11.11%^17^. However, as we did not perform DNA sequencing nor MLH1-hypermethylation assays, we could not be able to confirm the germline origin of MMRd. PTEN is another tumor suppressor gene involving various biological processes, including maintaining of genomic stability, cell survival, migration, proliferation, and metabolism^18^. As described in previous IHC series, we observed PTEN loss in more than 15% of cases^19,20^. High expression or amplification in HER proteins is frequent in OC and is associated with progression and prognosis^21^. Moreover, they can be targeted with dedicated therapies such as EGFR or HER2 inhibitors. EGFR and HER2 overexpression were found in a few cases in our set (16% and 9%, respectively). However, this was consistent with published data with a low frequency of ERBB2 overexpression/amplification in high-grade serous tumors^22^.

In this work, we have identified altered regions from other ethnicities that are very similar to what has been previously described in OC. A study carried out in 2018 identified alterations strongly associated with the disease, such as deletions at 1p36 and amplification at 8q24, 8p11, and 8q24.21^23^. It has also been shown that among genes involved in 20q13-amplification, *ADRM1* amplification was significantly upregulated concerning stage, recurrence, and metastasis^24^. Another German study in 2003 has clearly demonstrated that gains in DNA copy number were most often observed for *PIK3CA* on 3q (66%), *PAK1* on 11q (59%), 12p and 20q. The same genes were altered in our samples but with different frequencies. The same study showed an amplification in chromosomes11q13-q14 and deletion in 17q and 15q, similar to what we observed^25,26^.

One of the main aims of this study was to explore correlations between IHC expression and CNA alterations in the corresponding genes. High PD-L1 expression was associated with amplification of CD274 (OR = 4.97,*p* = 5.79E-02). PD-L1 CNAs, particularly PD-L1 copy number gains, represent frequent genetic alterations across many cancers, which influence PD-L1 expression levels, are associated with higher mutational loads, and may be exploitable as predictive biomarkers for immunotherapy regimens^27^. We also observed a correlation between *ERBB2* amplification (17q12) and ERBB2 protein expression (*p* = 4.03E-09). A study carried out in 2021 demonstrated that focal amplification of the 17q12 chromosomal region containing *ERBB*2, with at least six copies, was then confirmed by in situ hybridization^28^. *ERBB2* amplification in OC remains a rare event. In our study, we showed that the *ERBB2* amplification was found mostly in 6% of HGSC, which is consistent with the study of Kohei Nakamura et al. showing that *ERBB2* and *KRAS* amplification were detected in HGSC as actionable cancer-related genetic alterations^29^.We were not able to show a higher amplification rate in mucinous tumors as we included only 4 mucinous carcinomas in our cohort^30^.

Survival was correlated with clinical parameters and CNA signatures. The overall survival was significantly correlated with menopausal status and MSH2 protein expression. However, this should be confirmed as only one case had low MSH2 expression in our cohort. A study carried out in Norway demonstrated that a lifetime risk of ovarian cancer of around 10% and a 20% risk of death from ovarian cancer give a lifetime risk of death from ovarian cancer of around 2% in women with an MMR gene mutation^31^. The Gamma-H2AX protein expression was significantly correlated with the s7 signature (BRCA-related non-HRD). Consolidation of Gamma-H2AX in the chromatin at the dsDNA break site isa focus for HR repair machinery^32^. In addition, the association between individual CNA signature exposures and overall survival using a combined dataset of 111 diagnostic samples with clinical outcomes shows that the two most represented signatures were s1signature (MAPK pathway) and s3 signature (BRCA-related homologous recombination deficiency), this results are similar to the McIntyre ‘s study, carried out in 2018 in a population from United Kingdom^12^. In a recent study, authors demonstrated in a cohort of 276 women with relapsed high-grade serous EOC from the BriTROC-1 study that copy number signatures were strongly correlated with immune cell infiltration. The copy number signature was stable between diagnosis and relapse. Moreover, *CCNE1* amplification, *KRAS* amplification, and s1 signature were predictive of early platinum-resistant relapse^33^. These results are partially consistent with our findings since the s1 signature was correlated to prognosis. Future comparative studies are needed to evaluate the increase of genomic instability in the Tunisian cohorts after chemotherapy.

Our study presents several limitations, including the fact that it was a retrospective, monocentric study and that the number of cases was limited (*N* = 198). Due to the lack of available African data sets, we could not confirm these results in an independent cohort from the same geographic region. The protein expression study was carried out on TMA, which can lead to ignoring tumor heterogeneity by analyzing only small tumor regions. However, we tried to decrease this bias as much as possible by assessing protein expression in triplicate for each sample. Our study was also limited to 14 proteins of interest. We thus have ignored other proteins that may be involved in ovarian carcinoma development. Concerning DNA analyses, the average quality of DNA (extracted from FFPE tissue) precluded the ability to perform extensive DNA sequencing in our population. Additional DNA sequencing may identify new punctual mutations in ovarian cancer and could reinforce some IHC results, such as P53 status. Finally, knowledge of BRCA status and results of approved HRD assays would have been interesting to explore their correlation to protein expression. Unfortunately, such assays were not routinely performed in Tunisia during the inclusion period.

## Conclusion

In conclusion, we observed in this large cohort of patients from North Africa that clinical, pathological, and CNAs are similar to what has been published in patients of Caucasian ethnicity. Despite being the first study exploring protein and CNA profiles of OC in a North African population, these results should be confirmed in other independent series. Moreover, complementary analyses, including gene sequencing and homologous recombination repair characterization, are awaited to deeply explore the OC from North Africa.

### Methods

#### Patients’ selection and data collection

In this retrospective study, we included patients with primary ovarian carcinoma (according to the criteria of the World Health Organization (WHO) classification) collected at the Salah Azaiez Cancer Institute (SAI) of Tunis between 2000 and 2018. Formalin-fixed paraffin-embedded (FFPE) samples were all a pre-therapeutic, collected at first laparoscopic or open diagnostic surgery. First, we selected patients with both clinical data and formalin-fixed paraffin-embedded (FFPE) samples available. We collected data relating to age at diagnosis, menopausal status, FIGO stage at diagnosis, histological subtype, histological grade, presence of residual disease after surgery, progression-free survival (PFS), and overall survival (OS). Informed consent was signed by the patient according to the recommendations of the Biomedical Ethics Committee of the Institut Pasteur in Tunis (IPT).

### Immunohistochemical analysis

All samples were organized in a Tissue microarray (TMA) block; each case was represented by two or three cores, each 1 mm in diameter, depending on the availability of FFPE blocks. Protein expression was analyzed by IHC (immunohistochemistry) on 4 μm thick sections from a TMA block comprising 198 ovarian carcinomas. For most antibodies described in Table 5, protein labeling was performed using the DAKO Autostainer (Dako Cytomation, Copenhagen, Denmark) according to standard protocols. The Dako LSAB kit was used, and Dako Flex system (Dako) revealed the antigen using a peroxidase enzyme. Primary antibody dilutions and unmasking pH are given in Table 5. After binding of the secondary antibody to the primary antibody, revelation is achieved by catalysis of the substrate “Di-Amino-Benzidine” (DAB), which is used to reveal peroxidase activity or HRP binding to the DAB chromogen in the presence of the H2O2 substrate. PD-L1 and HER2 IHC were performed in the biopathology department at IPC (Institut Paoli-Calmettes). Gamma-H2AX antibody staining was performed on the DiscoveryXT research automated system at ICEP (IPC/CRCM Experimental Pathology platform) with incubation at 37 °C and a Multimer revelation system. To facilitate the reading of the immunolabelled slides, they were scanned using a Nanozoomer 2.0HT slide scanner (Hamamatsu, Japan). The virtual slides were read (percentage of labeled cells + intensity) by pathologists at the ISA in Tunis using the CaloPix software (Tribun Health, Paris, France). Each case has been read in triplicate. Positive expression of the MMR was calculated by summing the number of cells with a positive staining, dividing by the total number of tumor cells (viable), and multiplying by 100.

The threshold for positivity was set at 10% for hormone receptor expression^34^, mismatch repair proteins, and PTEN^19^. All tumors with at least 1% of positive cells were classified as PD-L1 or EGFR positive. The threshold was defined at 70% for p16 based on the most frequently used definition available in the literature: strong and diffuse nuclear and cytoplasmic staining in ≥ 70% of the tumor cells^35–40^. ERBB2 expression was assessed according to ASCO/CAP guidelines with dichotomization between cases with no or low (1+) expression and those with 2 + and 3 + scores^41^. Concerning P53, diffuse (at least 90%) aberrant nuclear positivity was considered as mutant. Cases with no P53 staining, i.e., 0%, were also considered mutant. The thresholds for Gamma-H2AX and EZH2 were based on the median expression in our set (10% and 15%, respectively).

**Table 5:** Antibodies, clones and dilutions used in IHC.

Antibody	Clone	distributor	Dilution (primary Ab)	pH (unmasking)	Automate
MLH1	ES05	Dako IR079	RTU	9	Dako
MSH2	FE11	Dako IR085	RTU	9	Dako
MSH6	EP49	Dako IR086	RTU	9	Dako
PMS2	EP51	Dako IR087	RTU	9	Dako
PD-L1	22C3	Dako M3653	1/50	6	DakoOmnis
ER	EP1	Dako IR084	RTU	9	Dako
PR	PgR636	Dako IR068	RTU	9	Dako
PTEN	6H2.1	Dako M3627	1/50	9	Dako
P53	DO7	Dako M7001	1/100	9	Dako
p16	IHC116	Gene AB	RTU	9	Dako
HER2	4B5	Roche	RTU	9	BenchMark ULTRA
EGFR	31G7	Zymed28-0005	1/20	6	Dako
EZH2	D2C9	Cell signaling Technologies	1/200	6	Dako
Gamma-H2AX	JBW301	Merck Millipore	1/2000	9	Discovery

Ab : antibody ; pH : potential hydrogen ; RTU : ready-to-use

### DNA extraction

DNA was extracted from FFPE samples. Tumor areas were defined by the ICEP pathologist on HES (hematoxylin, eosin, and saffron) slides. The number of white slides to be cut (10 μm each) was determined by the percentage of tumor cells and the surface area of the circled zone. according to the manufacturer’s instructions, DNA extraction from the paraffin blocks was performed using the Maxwell^®^ RSC DNA FFPE Kit. The DNA was then recovered and quantified spectrophotometrically (NanoDrop, ND-1000). Qubit quantification was then done to confirm the DNA’s quality by calculating the percentage of double-stranded DNA.

### Comparative genomic hybridization on microarray (CGH array)

CGH-array is based on the principle of hybridization of ordered probes on a solid support with labeled targets corresponding to an equimolar mixture of normal and tumor DNA, each labeled with a specific fluorochrome (Cyanine 5 and Cyanine 3), enabling the identification of gains/amplifications or losses/deletions.

Using CGH-array analyses we established a comprehensive genomic description, and we analyzed the copy number alterations (CNA) on DNA samples using high-resolution microarrays (Hu-244 A, Agilent Technologies, Massy, France). Copy number alterations were characterized using Agilent 4 × 180 K high-resolution comparative genomic hybridization microarray (aCGH) technology, as previously reported^42^.

All microarray probes were aligned to the Human Hg19 reference genome. For each gene, two thresholds were used (log2 ratio > |0.2| and |0.9|) to distinguish between low-amplitude alterations (gains and losses) and high-amplitude alterations (amplifications and deletions). To identify recurrent copy number alterations, we used the Genomic Identification of Significant Targets in Cancer (GISTIC) 2.0 algorithm calculated by multiple random iterations, with an amplification/deletion threshold > 0.9, confidence level of 0.90, and a corrected threshold probability q < 0.25.Genomic signatures exposure were explored according to a previously published algorithm^12^.

### Statistical analyzes

Fisher’s exact test (for categorical variables) and Wilcoxon test (for continuous variables) were used to compare descriptive items. Survival curves were estimated using the Kaplan-Meier method. Follow-up was estimated by the Kaplan-Meier method. Progression-free survival (PFS) was defined as the time from diagnosis to disease relapse, progression, or death, whatever the cause. Overall survival (OS) was defined as the time from diagnosis to death from any cause. Data concerning patients without disease progression or death at the last follow-up were censored. The prognostic impact of the initial histo-clinical characteristics (age, menopausal status, FIGO stage, grade, residual disease after surgery) was assessed by the Cox regression method in the univariate analysis and the p values estimated with the Wald test. All statistical tests were done bilaterally, with a significance level of *p* ≤ 0.05. All statistical analyses were performed with R 3.5.1 software (R Development Core Team (2009) R Foundation for Statistical Computing, Vienna, Austria).

## Electronic supplementary material

Below is the link to the electronic supplementary material.

Supplementary Material 1

Supplementary Material 2

Supplementary Material 3

Supplementary Material 4

Supplementary Material 5

## Data Availability

The datasets generated and/or analysed during the current study are not publicly available due to institutional recommendations but are available from the corresponding author on reasonable request.

## Declarations

### Approval for human experiments

Specimens for the EOC-TMA were gathered from the SAI. All patients willingly provided specimens, accompanied by written informed consent, and the study received approval from the Institutional Ethics Committee at SAI (Number 2150, September 2020). The methods employed adhered to pertinent guidelines and regulations. Our study adhered to the Code of Ethics of the World Medical Association (Declaration of Helsinki), as published in the British Medical Journal on July 18th, 1964.
