# Supplementary material for: Decoding the pathological and genomic profile of epithelial ovarian cancer
Source: Sci Rep. 2024 Nov 19;14:28573. doi: 10.1038/s41598-024-80030-z (PMC11577113; doi:10.1038/s41598-024-80030-z)
Supplement: Supplementary file 1 — Supplementary Material 1 [file 41598_2024_80030_MOESM1_ESM.docx]

**Supplementary Table S3: Correlations between proteins and genes copy number alterations**

| Symbol | IHC | p_amp | odds_ratio_amp | p_gain | odds_ratio_gain | p_del | odds_ratio_del | p_loss | odds_ratio_loss |
| --- | --- | --- | --- | --- | --- | --- | --- | --- | --- |
|  |  |  |  |  |  |  |  |  |  |
| *TP53* | *TP53_mean_perc* | 1,00E+00 | 1,26 | 1,00E+00 | 0,75 | 1,00E+00 | 0,62 | 5,20E-03 | 2,65 |
| *ESR1* | *RE_mean_perc* | 1,00E+00 | Inf | 6,95E-01 | 2,59 | 1,00E+00 | 0,00 | 3,32E-01 | 1,52 |
| *PGR* | *RP_mean_perc* | 1,00E+00 | 1,07 | 5,31E-01 | 1,24 | 1,00E+00 | 0,00 | 1,00E+00 | 0,77 |
| *CD274* | *PDL1_mean_perc* | 5,79E-02 | 4,97 | 1,91E-04 | 3,80 | 1,00E+00 | 0,00 | 1,48E-02 | 0,40 |
| *PTEN* | *PTEN_mean_perc* | 1,00E+00 | Inf | 4,21E-01 | 1,96 | 6,35E-02 | 0,09 | 3,94E-02 | 0,40 |
| *CDKN2A* | *P16_mean_perc* | 4,98E-01 | Inf | 7,32E-06 | 6,75 | 1,04E-02 | 0,00 | 3,78E-04 | 0,28 |
| *EGFR* | *EGFR_mean_perc* | 2,51E-01 | 2,68 | 8,14E-01 | 1,09 | 1,00E+00 | 0,00 | 1,00E+00 | 1,04 |
| *MSH2* | *MSH2_mean_perc* | 1,00E+00 | Inf | 1,00E+00 | Inf | 1,00E+00 | 0,00 | 1,00E+00 | Inf |
| *MSH6* | *MSH6_mean_perc* | 1,00E+00 | Inf | 6,08E-01 | 0,53 | 1,00E+00 | 0,00 | 1,00E+00 | Inf |
| *MLH1* | *MLH1_mean_perc* | 1,00E+00 | Inf | 3,64E-01 | 0,35 | 1,00E+00 | 0,00 | 5,78E-01 | Inf |
| *ERBB2* | *HER2_QS* | 1,67E-05 | 69,32 | 6,64E-06 | 23,61 | 1,00E+00 | 0,00 | 1,62E-03 | 0,16 |
| *EZH2* | *EZH2_mean_perc* | 6,29E-02 | 0,16 | 6,34E-01 | 0,83 | 1,00E+00 | 0,00 | 2,32E-02 | 3,84 |
| *BRCA1* | *Gamma-H2AX_mean_perc* | 1,00E+00 | 0,00 | 7,32E-01 | 0,73 | 1,00E+00 | 0,00 | 2,08E-02 | 2,10 |
| IHC : Immunohistochemistry, amp : amplification, del : deletion, p : pvalue | | | | | | | | | |

**Supplementary Table S5: Correlations between proteins expression and GISTIC regions**

| IHC | Gistic_Region | freq_alt_IHC_pos | freq_alt_IHC_neg | freq_alt2_IHC_pos | freq_alt2_IHC_neg | p1 | p2 | FDR1 | FDR2 | Diff_freq |
| --- | --- | --- | --- | --- | --- | --- | --- | --- | --- | --- |
| TP53_mean_perc | Amplification Peak 13 2p15 | 0,44 | 0,26 | 0,07 | 0,05 | 4,70E-03 | 5,41E-01 | 1,95E-01 | 1,00E+00 | 1,67 |
| TP53_mean_perc | Amplification Peak 15 2q24.2 | 0,52 | 0,33 | 0,09 | 0,06 | 3,62E-03 | 4,03E-01 | 1,83E-01 | 1,00E+00 | 1,57 |
| TP53_mean_perc | Amplification Peak 24 3q26.31 | 0,78 | 0,59 | 0,38 | 0,22 | 2,64E-04 | 9,27E-03 | 4,97E-02 | 1,00E+00 | 1,32 |
| TP53_mean_perc | Amplification Peak 86 19q13.12 | 0,39 | 0,19 | 0,09 | 0,05 | 1,06E-03 | 2,49E-01 | 9,40E-02 | 1,00E+00 | 2,01 |
| RE_mean_perc | Amplification Peak 18 3q13.2 | 0,54 | 0,27 | 0,09 | 0,03 | 5,90E-03 | 3,06E-01 | 2,24E-01 | 1,00E+00 | 1,97 |
| PDL1_mean_perc | Amplification Peak 46 9p24.2 | 0,40 | 0,14 | 0,13 | 0,06 | 1,91E-04 | 1,81E-01 | 4,34E-02 | 1,00E+00 | 2,87 |
| PDL1_mean_perc | Amplification Peak 47 9p22.2 | 0,35 | 0,14 | 0,10 | 0,08 | 3,48E-03 | 1,00E+00 | 1,83E-01 | 1,00E+00 | 2,47 |
| PDL1_mean_perc | Amplification Peak 48 9p21.3 | 0,36 | 0,16 | 0,08 | 0,11 | 4,47E-03 | 6,21E-01 | 1,94E-01 | 1,00E+00 | 2,30 |
| PDL1_mean_perc | Deletion Peak 15 4p16.2 | 0,42 | 0,58 | 0,00 | 0,00 | 5,70E-03 | 1,00E+00 | 2,22E-01 | 1,00E+00 | 0,72 |
| PTEN_mean_perc | Amplification Peak 44 8q24.21 | 0,69 | 0,39 | 0,33 | 0,19 | 1,57E-03 | 1,94E-01 | 1,17E-01 | 1,00E+00 | 1,79 |
| P16_mean_perc | Amplification Peak 2 1p34.3 | 0,35 | 0,11 | 0,06 | 0,03 | 2,07E-04 | 5,02E-01 | 4,34E-02 | 1,00E+00 | 3,23 |
| P16_mean_perc | Amplification Peak 4 1p31.3 | 0,56 | 0,33 | 0,10 | 0,01 | 4,15E-03 | 1,04E-02 | 1,85E-01 | 1,00E+00 | 1,68 |
| P16_mean_perc | Amplification Peak 9 1q42.3 | 0,57 | 0,32 | 0,13 | 0,12 | 1,52E-03 | 1,00E+00 | 1,17E-01 | 1,00E+00 | 1,77 |
| P16_mean_perc | Amplification Peak 12 2p16.1 | 0,42 | 0,20 | 0,13 | 0,05 | 4,02E-03 | 8,71E-02 | 1,85E-01 | 1,00E+00 | 2,05 |
| P16_mean_perc | Amplification Peak 19 3q22.1 | 0,50 | 0,27 | 0,12 | 0,01 | 2,32E-03 | 2,85E-03 | 1,50E-01 | 7,77E-01 | 1,86 |
| P16_mean_perc | Amplification Peak 20 3q22.3 | 0,52 | 0,27 | 0,19 | 0,03 | 8,52E-04 | 5,56E-04 | 8,58E-02 | 2,17E-01 | 1,94 |
| P16_mean_perc | Amplification Peak 21 3q23 | 0,72 | 0,46 | 0,19 | 0,10 | 5,54E-04 | 9,90E-02 | 6,88E-02 | 1,00E+00 | 1,57 |
| P16_mean_perc | Amplification Peak 22 3q26.1 | 0,81 | 0,60 | 0,44 | 0,14 | 4,83E-03 | 1,07E-05 | 1,95E-01 | 1,07E-02 | 1,34 |
| P16_mean_perc | Amplification Peak 23 3q26.2 | 0,87 | 0,63 | 0,49 | 0,19 | 4,02E-04 | 4,47E-05 | 6,46E-02 | 3,02E-02 | 1,37 |
| P16_mean_perc | Amplification Peak 24 3q26.31 | 0,84 | 0,53 | 0,43 | 0,16 | 6,36E-06 | 1,07E-04 | 6,04E-03 | 4,87E-02 | 1,59 |
| P16_mean_perc | Amplification Peak 25 3q29 | 0,60 | 0,33 | 0,21 | 0,12 | 5,25E-04 | 1,20E-01 | 6,88E-02 | 1,00E+00 | 1,81 |
| P16_mean_perc | Amplification Peak 46 9p24.2 | 0,35 | 0,12 | 0,10 | 0,06 | 4,68E-04 | 4,43E-01 | 6,88E-02 | 1,00E+00 | 2,93 |
| P16_mean_perc | Amplification Peak 47 9p22.2 | 0,32 | 0,12 | 0,10 | 0,08 | 2,38E-03 | 6,20E-01 | 1,50E-01 | 1,00E+00 | 2,67 |
| P16_mean_perc | Amplification Peak 48 9p21.3 | 0,37 | 0,10 | 0,12 | 0,08 | 2,36E-05 | 3,45E-01 | 1,07E-02 | 1,00E+00 | 3,80 |
| EGFR_mean_perc | Deletion Peak 47 10q23.2 | 0,19 | 0,48 | 0,00 | 0,00 | 2,12E-03 | 1,00E+00 | 1,48E-01 | 1,00E+00 | 0,40 |
| HER2_QS | Amplification Peak 77 17q12 | 0,41 | 0,03 | 0,29 | 0,01 | 6,64E-06 | 5,53E-05 | 6,04E-03 | 3,02E-02 | 14,58 |
| HER2_QS | Deletion Peak 23 5q14.1 | 0,18 | 0,53 | 0,00 | 0,00 | 6,86E-03 | 1,00E+00 | 2,46E-01 | 1,00E+00 | 0,34 |
| HER2_QS | Deletion Peak 76 17q11.2 | 0,24 | 0,68 | 0,00 | 0,00 | 2,01E-04 | 1,00E+00 | 4,34E-02 | 1,00E+00 | 0,35 |
| HER2_QS | Deletion Peak 77 17q12 | 0,41 | 0,73 | 0,00 | 0,00 | 3,59E-03 | 1,00E+00 | 1,83E-01 | 1,00E+00 | 0,56 |
| CLASSIF_ERBB2 | Amplification Peak 77 17q12 | 1,00 | 0,04 | 1,00 | 0,01 | 5,32E-07 | 1,41E-08 | 1,45E-03 | 3,85E-05 | 26,86 |
| EZH2_mean_perc | Amplification Peak 2 1p34.3 | 0,32 | 0,14 | 0,06 | 0,03 | 5,70E-03 | 4,97E-01 | 2,22E-01 | 1,00E+00 | 2,26 |
| EZH2_mean_perc | Amplification Peak 5 1p13.2 | 0,51 | 0,28 | 0,16 | 0,09 | 1,58E-03 | 2,74E-01 | 1,17E-01 | 1,00E+00 | 1,85 |
| EZH2_mean_perc | Amplification Peak 12 2p16.1 | 0,43 | 0,19 | 0,09 | 0,09 | 8,80E-04 | 1,00E+00 | 8,58E-02 | 1,00E+00 | 2,20 |
| EZH2_mean_perc | Amplification Peak 56 11q13.2 | 0,41 | 0,19 | 0,07 | 0,07 | 2,42E-03 | 1,00E+00 | 1,50E-01 | 1,00E+00 | 2,10 |
| EZH2_mean_perc | Amplification Peak 65 12q12 | 0,49 | 0,27 | 0,08 | 0,07 | 2,47E-03 | 1,00E+00 | 1,50E-01 | 1,00E+00 | 1,85 |
| EZH2_mean_perc | Amplification Peak 86 19q13.12 | 0,39 | 0,18 | 0,10 | 0,03 | 3,54E-03 | 8,09E-02 | 1,83E-01 | 1,00E+00 | 2,10 |
| YH2AX_mean_perc | Amplification Peak 44 8q24.21 | 0,82 | 0,52 | 0,45 | 0,20 | 1,26E-04 | 1,27E-03 | 3,44E-02 | 3,85E-01 | 1,57 |
| YH2AX_mean_perc | Amplification Peak 45 8q24.3 | 0,77 | 0,53 | 0,45 | 0,14 | 3,45E-03 | 1,18E-05 | 1,83E-01 | 1,07E-02 | 1,45 |
| YH2AX_mean_perc | Amplification Peak 57 11q14.1 | 0,43 | 0,21 | 0,12 | 0,06 | 3,78E-03 | 1,88E-01 | 1,84E-01 | 1,00E+00 | 2,06 |
| YH2AX_mean_perc | Deletion Peak 53 11q23.3 | 0,18 | 0,39 | 0,00 | 0,00 | 6,74E-04 | 1,00E+00 | 7,36E-02 | 1,00E+00 | 0,47 |
| IHC : Immunohistochemistry, freq_alt_IHC_pos : frequency of alteration of positive IHC, freq_alt_IHC_neg : frequency of alteration of negative IHC, p : pvalue | | | | | | | | | | |
