# Supplementary material for: Decoding the pathological and genomic profile of epithelial ovarian cancer
Source: Sci Rep. 2024 Nov 19;14:28573. doi: 10.1038/s41598-024-80030-z (PMC11577113; doi:10.1038/s41598-024-80030-z)
Supplement: Supplementary file 5 — Supplementary Material 5 [file 41598_2024_80030_MOESM5_ESM.docx]

**Supplementary Figure S4: Association between TP53 expression and CGH signatures**

**
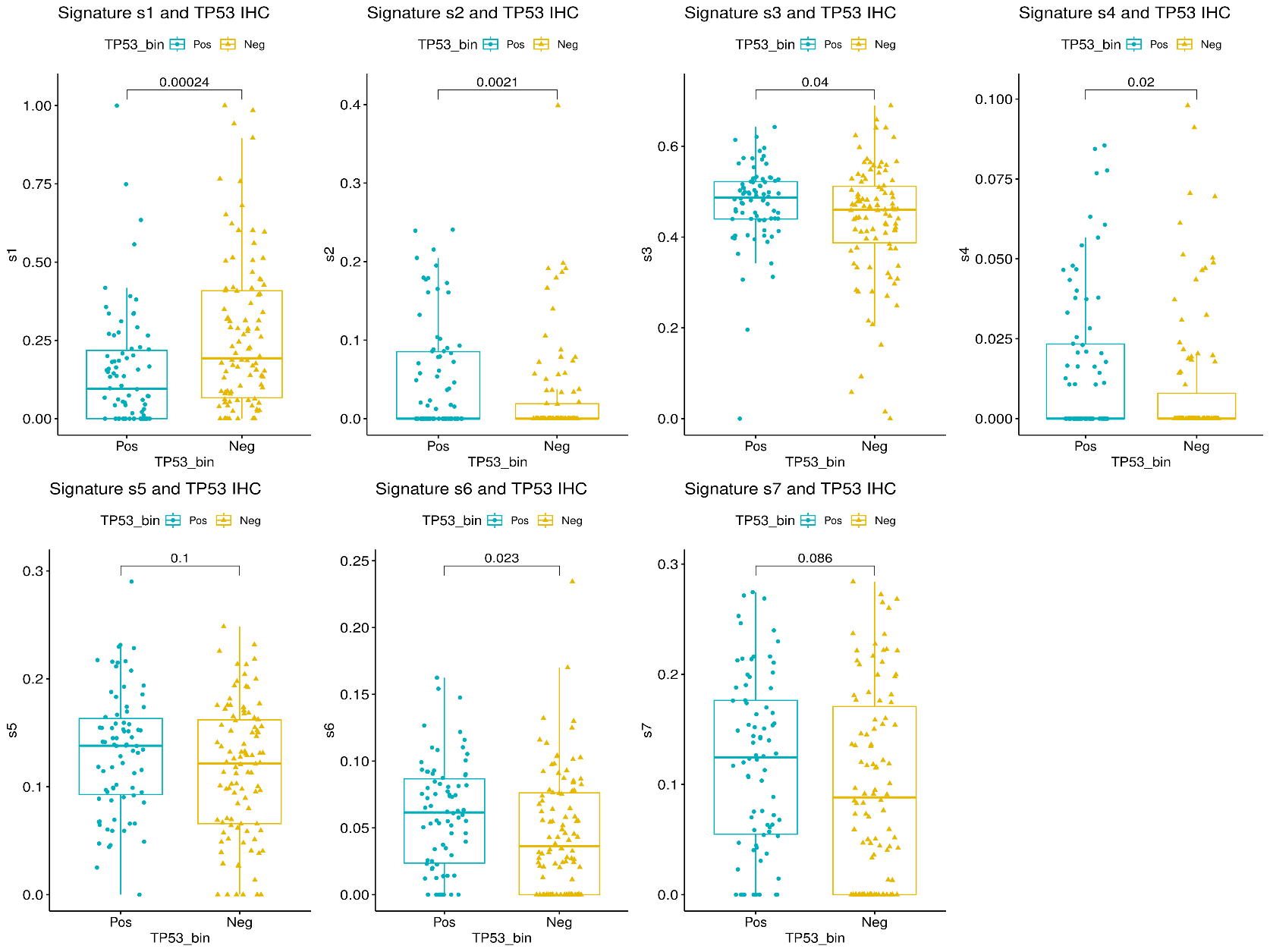
**

**Supplementary Figure S4: Association between TP16 expression and CGH signatures**


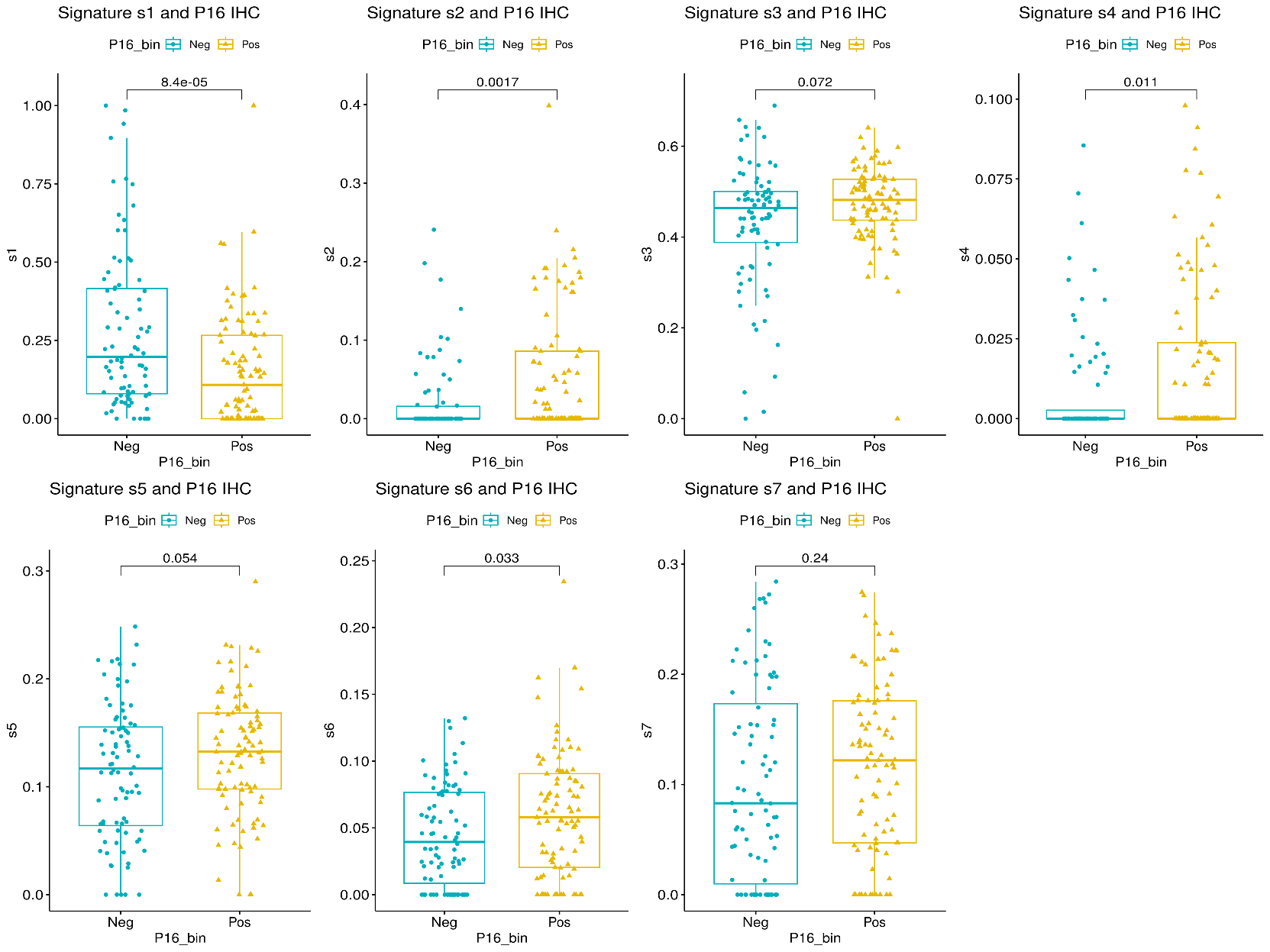


**Supplementary Figure S4: Association between EZH2 expression and CGH signatures**

**
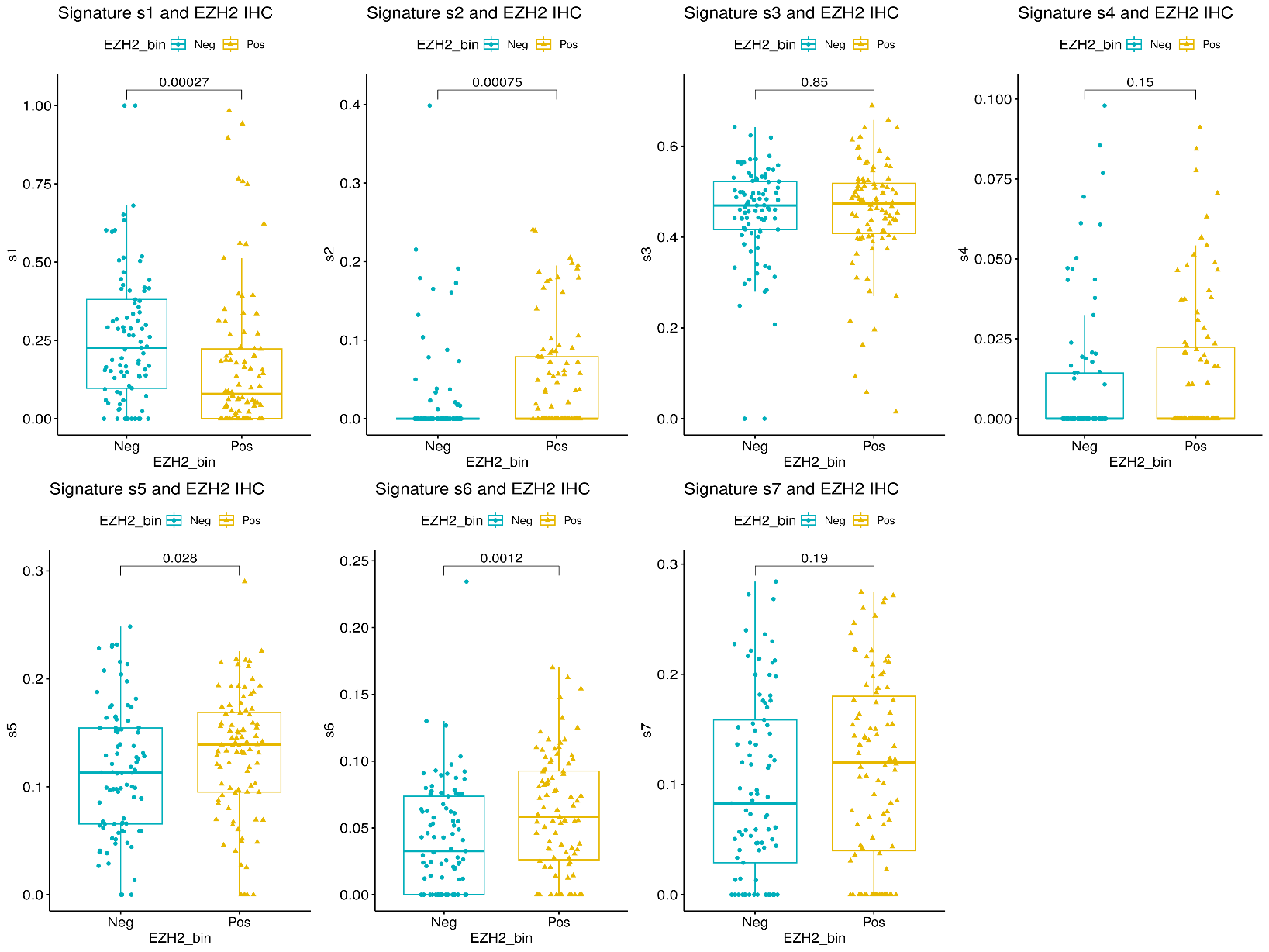
**

**Supplementary Figure S4: Association between Gamma-H2AX expression and CGH signatures**

**
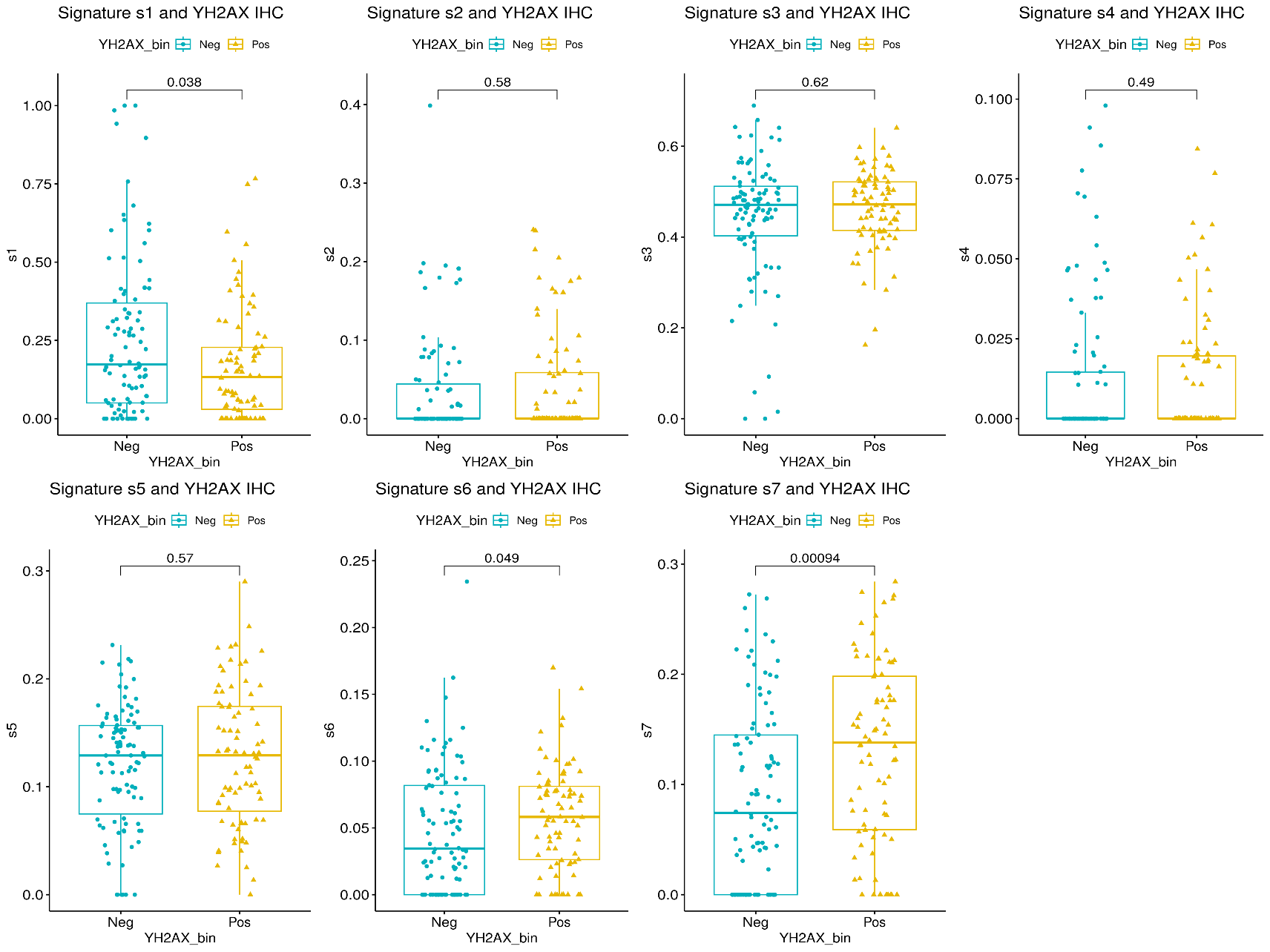
**
